# Diabetes and Phacoemulsification Cataract Surgery: Difficulties, Risks and Potential Complications

**DOI:** 10.3390/jcm8050716

**Published:** 2019-05-20

**Authors:** Andrzej Grzybowski, Piotr Kanclerz, Valentín Huerva, Francisco J. Ascaso, Raimo Tuuminen

**Affiliations:** 1 Department of Ophthalmology, University of Warmia and Mazury, 10-082 Olsztyn, Poland; 2 Institute for Research in Ophthalmology, Foundation for Ophthalmology Development, 60-554 Poznan, Poland; 3 Hygeia Clinic, 80-286 Gdańsk, Poland; p.kanclerz@gumed.edu.pl; 4 Department of Ophthalmology, University Hospital Arnau de Vilanova, 25198 Lleida, Spain; vhuerva@gmail.com; 5 Department of Ophthalmology, Hospital Clínico Universitario Lozano Blesa, 50009 Zaragoza, Spain; jascaso@gmail.com; 6 Aragon Health Research Institute (IIS Aragon), 50009 Zaragoza, Spain; 7 Helsinki Retina Research Group, Faculty of Medicine, University of Helsinki, 00014 Helsinki, Finland; raimo.tuuminen@helsinki.fi; 8 Department of Ophthalmology, Kymenlaakso Central Hospital, 48210 Kotka, Finland

**Keywords:** cataract surgery, diabetic macular edema, diabetes mellitus, diabetic retinopathy, phacoemulsification

## Abstract

Diabetes mellitus is one of the most prevalent chronic diseases worldwide. Diabetic patients are at risk of developing cataract and present for surgery at an earlier age than non-diabetics. The aim of this study was to review the problems associated with cataract surgery in a diabetic patient. Corneal complications in diabetic patients include delayed wound healing, risk of developing epithelial defects or recurrent erosions due to the impairment of epithelial basement membranes and epithelial–stromal interactions. Diabetic patients present lower endothelial cell density and their endothelium is more susceptible to trauma associated with cataract surgery. A small pupil is common in diabetic patients making cataract surgery technically challenging. Finally diabetic patients have an increased risk for developing postoperative pseudophakic cystoid macular edema, posterior capsule opacification or endophthalmitis. In patients with pre-proliferative or proliferative diabetic retinopathy, diabetic macular edema or iris neovascularization adjunctive therapy such as an intravitreal anti-vascular endothelial growth factor injection, can inhibit exacerbation related to cataract surgery.

## 1. Introduction

Diabetes mellitus (DM) is one of the most prevalent and morbid chronic diseases, and affects millions of patients worldwide. As diabetic patients have an increased risk of developing cataract, particularly being susceptible to develop cortical and posterior subcapsular opacities, they present for surgery at an earlier age [1]. The relative risk of developing cataract increases with the duration of diabetes, severity of hyperglycemia and age [2]. Accumulation of advanced glycation end products (AGE) in the lens is one of the mechanisms of diabetic cataract development [3].

Cataract surgery in diabetic patients carries a higher risk of both intraoperative and postoperative complications compared to non-diabetic patients [4,5]. The aim of this study was to review the problems associated with cataract surgery in diabetic patients.

## 2. Results

We identified 116 eligible publications from the PubMed and Web of Science database (the search strategy in detail is presented in Supplement flie S1). Emphasis was placed on articles published since the review by Peterson et al. [6], but we included earlier articles that provided a more comprehensive view. 

### Alterations in Refraction and Biometry in Diabetic Patients

Patients with DM present variations in refraction associated with changes of blood glucose levels. This effect is particularly manifested in patients receiving improved control after hyperglycaemia, who might develop a hyperopic change ranging from 0.5 Diopters (D) to 3.75 D for 1–2 weeks after initiation of treatment [7]. During hyperglycemia, excess glucose in the lens is converted by aldose reductase to sorbitol. Experimental studies have shown that osmotic gradient across an intact capsule is possible [8]. With rapid changes from a hyper- to hypo-glycemic state, glucose can easily migrate from the lens while sorbitol cannot. The differences in osmotic pressure result in the influx of aqueous humor and lens swelling. Histological studies have shown that lenses of diabetic rats manifest vacuoles in the periphery [8]. As cataract develops, the vacuoles were also found in the anterior surface, resulting in cortical opacities. Peripheral lens fiber cells become swollen in contrast to those in deeper layers [8]. Modification of lens thickness due to changes in glycemic levels was not confirmed in objective measurements. The alterations in refraction are attributed to changes in the lens’s refractive index [7]. Although the changes in refraction should prompt strict glucose control, an advantage of cataract surgery is that variations in refraction will be eliminated.

In some cases the reliability of biometry in diabetic patients might be lower than in non-diabetics. An increase in central corneal thickness was reported in diabetic eyes, which could alter corneal power [9]. Sonmez et al. reported topographic changes of the flattest corneal meridian after intensive glucose control [10]. In a study by Adnan et al., patients with type 1 DM had greater lens thickness and lower anterior chamber depth compared to normal controls [11]. In patients with clinically significant macular edema A-scan measurements differ significantly from those of IOLMaster (v 3.01.0294) resulting in axial length estimation error [12]. The discrepancy in axial length was attributed to differences in the methodology of measuring the pathologically thickened retina between devices; it was concluded that IOLMaster might be less affected. Diabetic eyes demonstrate alterations of the vitreomacular interface, particularly thickening of the premacular cortical vitreous [13]. In another study, the IOLMaster (v 3.01.0294) was not able to differentiate the dense posterior vitreous membrane from the anterior surface of the macular edema [14].

The significance of blood glucose levels on biometry should not be overestimated. Biometry in diabetic eyes should be performed with caution, preferably with the possibility to review the method for assessing the posterior vitreous and macula. There is no direct evidence that diabetes or changes in blood glucose levels influence the final outcome of biometry. 

## 3. Diabetic Cornea in Cataract Surgery

### 3.1. Dry Eye and Epithelial Healing

DM is one of the major risk factors for dry eye syndrome [15]. In a hospital-based study, up to 54.0% of diabetic patients suffered from dry eye syndrome [16]. Development of dry eye symptoms in diabetics is associated with diabetic neuropathy, particularly changes in innervation and corneal hypoesthesia. In vivo studies have shown that patients with diabetic retinopathy (DR) have a lower density and greater tortuosity of nerves in the sub-basal plexus compared to normal eyes [17]. Conjunctival impression cytology demonstrates a significantly higher grade of squamous metaplasia and lower goblet cell density in diabetic eyes. Moreover, long-term hyperglycemia results in lacrimal gland dysfunction [18]. Studies demonstrated that lacrimal glands’ weight and tear film volume are lower in diabetic rats compared to controls [19]. Diabetic tear film has a reduced lipid layer and manifests lower stability [18].

Delayed corneal wound closure was demonstrated on diabetic rats [20]. Diabetic patients manifest have impairment of the epithelial basement membrane and epithelial-stromal interactions [21]. Due to epithelial dysfunction, diabetic corneas have a greater risk of developing epithelial defects, recurrent erosions, superficial punctate keratopathy, decreased sensitivity, delayed epithelialization, abnormal wound repair, increased susceptibility to injury, and ulceration [22,23]. Epithelial lesions are found in up to 64% of diabetic patients and are more common in diabetes mellitus type 2 than in type 1 [24]. Diabetic patients manifest impaired growth factor production, altered angiogenic response, macrophage function, collagen accumulation, in addition to keratinocyte and fibroblast migration and proliferation [25], which could partially contribute to inappropriate corneal wound healing. Corneal abrasions developed during or after surgery might present delayed healing or lead to recurrent corneal erosions [26]. A case report of complete bullous epithelium detachment due to basement membrane abnormalities during cataract surgery in a diabetic patient was reported [27]. Although there is no data on corneal wound healing after cataract surgery, the procedure should be as atraumatic as possible for the cornea, and special care should be taken to protect the epithelium. 

Cataract surgery can exacerbate a pre-existing dry eye disease, and the surgery has a potential to induce dryness in previously healthy eyes [28,29]. In a study by Ishrat et al., 64.3% of patients after cataract surgery had mild eye dryness [30]. Development of dry eye symptoms after surgery is associated with the mechanical trauma induced by surgery, exposure to microscopic light and tear film instability [31,32,33]. The symptoms are usually transient, and have a tendency to resolve in up to 3 months after surgery. Interestingly, in the study by Liu et al. cataract surgery induced dry eye symptoms both in diabetic and non-diabetic patients, however, corneal fluorescence staining returned to preoperative levels only in non-diabetic subjects [34]. In another study, 17.1% of diabetic patients and only 8.1% of non-diabetic patients developed dry eye syndrome 7 days after surgery [35]. In all individuals, symptoms resolved 3 months postoperatively, however, in diabetic patients the recovery occurred significantly slower.

### 3.2. Risk of Endothelial Damage

Diabetic patients present lower endothelial cell density compared to non-diabetic individuals of the same age [36,37]. The endothelial cell count inversely correlates with the duration of diabetes [36] and levels of Hemoglobin A1c (HbA1c) [38], and is lower in diabetes type 1 than type 2 [39]. Other studies reported a lower percentage of hexagonal cells and polymegathysm in diabetic corneas [40,41,42], as well as higher values of central corneal thickness compared to non-diabetics [9].

In diabetic patients, endothelium might be more susceptible to trauma and have weaker compensatory capabilities. Cataract surgery in diabetic patients results in greater endothelial cell loss compared to non-diabetics [43,44,45,46], as well as a decrease is hexagonal cells count [44,46]. Lower endothelial cell count was also found in manual small incision cataract surgery [47]. The harm associated with lens surgery in diabetic patients might be a result of reduced Na^+^–K^+^–ATPase activity influencing endothelial cell metabolism [48]. Furthermore, as glucose concentrations in aqueous humor might be frequently increased in diabetics leading to metabolic acidosis of the corneal matrix and decreased repairing capacity [45]. Another hypothetical explanation is the more common surgically induced miosis in diabetic eyes and the necessity to perform phacoemulsification closer to the cornea [49]. Finally, as diabetic patients manifest lower sub-basal nerve plexus density than non-diabetics, and cataract surgery furthermore reduces its’ density, diabetic patients are predisposed to develop diabetic keratopathy [50]. Persistent corneal edema after cataract surgery is uncommon. However, it was reported to occur more frequently in diabetics than in non-diabetics [51].

## 4. Risk of Infections in Diabetes

### 4.1. Conjunctivitis and Blepharitis

Diabetics have a higher prevalence of conjunctival colonization with *Staphylococcus aureus*, *Enterococci*, some forms of *Streptococci* and *Kliebsiella* spp. than non-diabetics [52]. Advanced age and abnormally high blood creatinine level was found to be associated with increased conjunctival colonization both in diabetics and non-diabetics [52]. The rate of conjunctival colonization is also correlated with the severity of DR [53]. A study by Kruse et al. demonstrated that the overall odds ratio of developing acute infectious conjunctivitis in individuals with diabetes is 1.24 (95% CI: 1.13–1.38) [54]. The increased prevalence might be associated with the hyperglycaemic environment encouraging bacterial colonization and disruption of normal conjunctival flora more frequently than in non-diabetic individuals’ antibiotic therapy [54]. The rate of conjunctival colonization in diabetic patients emphasizes the need for proper perioperative antisepsis in cataract surgery [55].

The pathophysiology of blepharitis involves several interactions including abnormal eyelid margin secretions, microbial infestation, and abnormalities of the tear film [56]. Given the aforementioned facts, it might be speculated that diabetes is a blepharitis predisposing factor [57].

### 4.2. Endophthalmitis

In general, diabetics have an increased risk of developing systemic infections, particularly pneumonia, urinary infection, wound infection, and bacteremia. Hyperglycemic environments lead to immune dysfunction including damage to the neutrophil function, depression of the antioxidant system, and decreased humoral immunity [58]. A better control of DM improves the aforementioned cellular functions [59,60]. Some microorganisms, i.e., *Candida albicans* were shown to manifest increased adherence to cells of diabetic patients, and became more virulent in a high glucose environment [59]. Jabbarvand et al. presented that the odds ratio for developing endophthalmitis in diabetic patients is 2.92 (95% CI: 1.72–4.96) [61].

Another problem for diabetic patients is impaired wound healing; the corneal wound may be predisposed to disintegration and persistent wound defects. As mentioned previously, conjunctival colonization rates are higher in diabetics than in non-diabetic individuals. It is not surprising that diabetic patients show an increased risk of developing endophthalmitis [62,63,64]. Moreover, endophthalmitis in diabetic patients is associated with poorer visual outcomes [65]. In the Endophthalmitis Vitrectomy Study, only 39% of diabetics achieved a visual acuity of 20/40, compared to 55% of the non-diabetic cohort [65].

## 5. Pupil Size in Diabetes

Disorders frequently seen in diabetics are small pupil size, light reflex disorder, and difficulties in dilation with commonly used mydriatics [66]. Alterations in pupillary function are mainly due to autonomic neuropathy, which predominantly involves the sympathetic innervation of the iris dilator [67]. Parasympathetic innervation of the iris sphincter is relatively spared. The loss of sympathetic tonus in diabetics limits the usefulness of commonly used anticholinergic agents, resulting in poor pupil dilation [68]. The addition of directly acting sympathomimetic, to which the pupil is hypersensitive, may provide sufficient mydriasis [69]. Importantly, sympathetic denervation is directly related to the duration of disease and to the development of systemic autonomic neuropathy [70]. In diabetic patients’ eyes previously treated with retinal laser photocoagulation, the pupils dilated less compared to eyes not treated with laser [69]. 

A small pupil makes cataract surgery technically challenging, and usually a stepwise approach for pupil dilation is recommended. In several cases, mydriasis might be achieved by the preoperative application of topical phenylephrine [69]. A 10% concentration is recommended, particularly in cases with darkly pigmented iris [71]. In addition, intracameral sympathomimetics agents (e.g., epinephrine in a 1:2500 dilution) can be administered intraoperatively [72]. The mydriatic effect might be also achieved with intracameral preservative-free lidocaine 1% [73,74]. In the study by Joshi intracameral lidocaine 1% provided sufficient mydriasis in type 2 diabetic patients undergoing cataract surgery [75]. Lidocaine might be combined with sympathomimetics and/or tropicamide. Such a preparation for intracameral administration is available commercially (Mydrane, Thea Pharmaceuticals, Clermont-Ferrand, France). In moderate to severe small pupil cases it might be required to mechanically stretch the iris. Manual pupil stretching should be avoided in eyes with iris neovascularization as the fragile vessels can rupture and cause hyphema or intraocular bleeding. Another option is employing iris hooks or pupil expansion devices, e.g., a Malyugin ring (Microsurgical Technology, Redmond, WA, USA), an I-Ring (Beaver Visitec International, Waltham, MA, USA), an Oasis (Oasis Medical Inc., Glendora, CA, USA), a B-Hex (MedInvent Devices, Kolkata, India), or APX-200 pupil expander (FCI Ophthalmics, Pembroke, MA, USA) [76,77,78,79,80].

Diabetic patients manifest decreased pupil size up to 1 months after cataract surgery, which might partially be explained by postoperative inflammation [81]. Nevertheless, the pupil area recovers to preoperative size 3 months after surgery.

## 6. Iris and Iridocorneal Angle Neovascularization

Aggressive treatment of neovascular glaucoma should take priority over cataract surgery because prolonged increase in intraocular pressure (IOP) can cause permanent damage to the optic nerve. Cataract surgery may exacerbate the neovascularization. Intravitreal anti-vascular endothelial growth factor (VEGF) therapy is the key strategy for short-term neovascular glaucoma management [82]. Anti-VEGF treatment results in direct decrease of intraocular VEGF levels and causes regression of neovascularization in the retina, iridocorneal angle, and iris. Anti-VEGF agents are administered usually before cataract surgery, during cataract surgery and after it [83].

## 7. Complications of Cataract Surgery in Diabetic Patients

### 7.1. Pseudophakic Cystoid Macular Edema—Prophylaxis Severity of Diabetes in Terms of Prophylaxis

Chu et al. revealed that diabetes is a risk factor for developing postsurgical pseudophakic cystoid macular edema (PCME) [84]. More importantly, the relative risk for developing PCME was associated with the staging of DR; it was 1.8 (95% confidence interval 1.36–2.36) in diabetic patients with no signs of retinopathy, 6.23 (95% CI: 5.12–7.58) in those with non-proliferative DR, and 10.34 (95% CI: 5.13–20.85) in individuals with proliferative diabetic retinopathy (PDR) [85]. In the study by Yang et al., the duration, severity, type of diabetes, as well as the hardness of the lens, and HbA1c levels were risk factors for development of PCME after cataract surgery in diabetic patients [86]. These findings indicate that diabetic patients should receive prompt PCME prophylaxis depending on the staging of DR. In patients requiring panretinal photocoagulation it was found that the best corrected-visual acuity (BCVA) and progression of macular edema was decreased if cataract surgery was performed prior to photocoagulation [87].

Singh et al. presented that a significantly lower percentage of diabetic patients receiving topical nepafenac (0.1%) and prednisolone acetate (1.0%) developed PCME compared to those who received solely prednisolone acetate [88]. The Royal College of Ophthalmologists recommends the use of a topical nonsteroidal anti-inflammatory drugs (NSAIDs) in addition to a steroid in patients with increased risk of PCME, e.g., in diabetes, with previous CME, previous retinal vein occlusion or epiretinal membrane [89]. The Prevention of Macular Edema (PREMED) study revealed that an subconjunctival injection of 40 mg triamcinolone acetonide (TA) at the conclusion of cataract surgery decreased the postoperative macular thickness if applied additionally to the standard regimen of bromfenac 0.09% eye drops twice daily (2 days preoperatively and 2 weeks postoperatively), and dexamethasone phosphate 0.1% (4 times a day for 2 days preoperatively and 1 week postoperatively, and then 1 drop less per day every subsequent week) [90]. Nevertheless, such treatment was not free of risks; 7.1% of patients treated with 40 mg of TA manifested IOP of 25 mm Hg or higher within 12 weeks. 

It can be concluded that patients with risk factors should receive a topical NSAID and a potent lipophilic steroid postoperatively, preferably with a subconjunctival injection of TA at the end of surgery.

### 7.2. The Influence of the Type, Severity, and Metabolic Compensation of Diabetes on the Risk of Complications

There is evidence that intensive diabetes treatment reduces long-term risk of undergoing any ocular surgery in individuals with type 1 diabetes [91]. Ylinen et al. reported that poor glycaemic control and insulin dependence are associated with postoperative central retinal thickness increase [92]. Paradoxically, Suto et al. presented that patients with rapid glycaemic correction in the three months before surgery had increased risk of postoperative progression of retinopathy and maculopathy [93]. In a survey by Woo et al., 86.0–93.8% of respondents would adopt a treat and defer strategy with preoperative blood glucose level over 305 mg/dL and 86.0–96.8% would cancel surgery with a blood glucose level of 410 mg/dL [94].

Development of posterior capsular opacification and continuous postoperative inflammation is more common in diabetics. Even in an uncomplicated cataract surgery, as DR can exacerbate during the postoperative period, patients should be monitored closely with ophthalmic examination. Modjtahedi et al. presented that patients with higher HbA1c concentrations had more severe retinal complications [85]. With that, the risk for developing PCME is associated with poor glycemic control, determined by the high serum HbA1c level [92].

### 7.3. Cataract Surgery in Patients with Diabetic Macular Edema (DME)

The PREMED study did not determine an effect of intravitreal 1.25 mg bevacizumab during cataract surgery on the final macular thickness in a general cohort of diabetic patients [90]. However, several studies have addressed the influence of anti-VEGF therapy among patients with DME undergoing cataract surgery. In a study by Takamura et al., an intravitreal injection of 1.25 mg bevacizumab not only prevented an increase in macular thickness after cataract surgery, but also resulted in reduction of macular thickness [95]. In a study by Lanzagorta-Aresti and associates, intravitreal bevacizumab immediately after phacoemulsification in DME eyes prevented exacerbation of macular edema compared to eyes that received a balanced salt solution [96].

Intravitreal agents are commonly administered at the conclusion of surgery through a pars plana approach, while some authors proposed intravitreal administration at the beginning of cataract surgery [97]. Others postulated injection through a paracentesis and a minute hole in the posterior capsule created by the 32-gauge needle after intraocular lens (IOL) insertion or trans-zonular approach [98,99].

Although anti-VEGF agents remain the first-line treatment for patients with DME, intravitreal corticosteroids have a substantial role as well [100]. In general, intravitreal corticosteroids are considered more appropriate for pseudophakic patients or for those being considered for cataract surgery in the near future, as their application is associated with risk of cataract progression [100]. Lim et al. compared the visual outcome and central macular thickness between intravitreal bevacizumab 1.25 mg and intravitreal triamcinolone (IVT) 4 mg administered at the time of cataract surgery for DME [101]. Both groups gained vision and had a reduction in retinal thickness, however, only IVT treatment resulted in a sustained reduction of macular thickness for six months after surgery. Similar results were reported after implantation of a sustained-release 700 μg dexamethasone intravitreal implant (Ozurdex, Allergan Inc, Irvine, CA, USA) [102]. Although IOP elevation is a well known complication of intravitreal steroids, none of the aforementioned studies reported a significant IOP increase.

Diabetic patients have a substantial risk of developing DME after cataract surgery, and particularly in the 3–6 months postoperative period [103]. The risk of developing DME in the first year after surgery is associated with the grading of retinopathy. In a study by Denniston et al., the risk was 1.0% for patients with no DR, 5.4% for those with mild non-proliferative diabetic retinopathy (NPDR), 10.0% in moderate NPDR, 13.1% in severe NPDR, and 4.9% in PDR [103].

### 7.4. Cataract Surgery in PDR—the Role of Anti-VEGF Agents

Several studies reported progression of DR after cataract surgery. Jaffe et al. noted progression in 70% of individuals after extracapsular cataract extraction [104], while Squirrel et al. a rate of 20% after phacoemulsification cataract surgery [105]. The rate of progression is influenced by the preoperative stage of DR, duration of DM, and glycaemic control [106]. It is postulated that physical trauma related to surgical manipulations within the anterior chamber induces an inflammatory response. The release of arachidonic acid from uveal tissue results in the production of leukotrienes (via the lipoxygenase pathway) and/or prostaglandins (via the cyclooxygenase pathway). These mediators of inflammation diffuse posteriorly and are responsible for progression of DR and ensue in the disruption of the blood–aqueous barrier with subsequent DME development.

Chema et al. reported that an intravitreal injection of 1.25 mg bevacizumab at the end of surgery prevented development of DR after surgery; DR it was observed in 11% of treated eyes postoperatively, compared to 45% in the control group [107].

### 7.5. Postoperative Posterior Capsule Opacification (PCO) and Intraocular Lens Biocompatibility

Diabetic patients are at risk of developing posterior capsule opacification after cataract surgery [108]. The difference in PCO rates after cataract surgery with hydrophobic IOL implantation in diabetic patients and non-diabetics was observed at 6 months after surgery and later [109], at 12 months [110], and over 18 months [108]. In the study by Hayashi et al., the systemic status, medical treatment of diabetes, or duration of the disease did not correlate with the degree of PCO [108]. In the study by Praveen et al. the risk of PCO was associated with the duration of diabetes [110]. Moreover, hydrophilic IOLs might be more prone to calcification [111]. Thus, implantation of hydrophobic IOLs might be recommended in diabetic patients [112].

## 8. Visual Outcome of Cataract Surgery in Diabetic Patients

In a study by Eriksson et al., patients with mild to moderate retinopathy, and without previous macular edema, had the same visual outcome of cataract surgery at 6 months as non-diabetic patients [113]. However, individuals with diabetes presented increased frequency of macular changes, and thus significantly worse visual acuity 6 weeks after surgery. In another study, BCVA increased in all diabetic patients regardless of the degree of diabetic retinopathy [114]. In a study by Stunf Pukl et al., both diabetics without DR and non-diabetics manifested a similar improvement in BCVA [115]. Diabetic patients had a slight decrease in retinal sensitivity, which could correspond to greater macular thickness. A recent study by Liu et al. presented that patients with diabetic retinopathy are less likely to achieve a 20/20 BCVA after cataract surgery compared to those without diabetes [116]. However, diabetic patients gained as many lines of BCVA as non-diabetics [116].

## 9. Conclusions

Cataract surgery in a diabetic patient is associated with several difficulties (Table 1). Diabetic patients present lower endothelial cell density and their endothelium is more susceptible to trauma associated with surgery. A small pupil is common in diabetic patients making surgery technically challenging. Finally diabetic patients have an increased risk for developing postoperative pseudophakic cystoid macular edema, posterior capsule opacification, or endophthalmitis. In patients with severe non-proliferative, proliferative diabetic retinopathy, diabetic macular edema or iris neovascularization adjunctive therapy such as an intravitreal anti-VEGF injection, may inhibit exacerbation related to cataract surgery. 

## Figures and Tables

**Table 1:** Problems associated with cataract surgery in a diabetic patient.

Pre- and Intraoperative	Postoperative
greater endothelial cell loss	increased risk of pseudophakic cystoid macular edema
higher prevalence of conjunctival colonization and supposedly blepharitis	increased risk of postoperative endophthalmitis
difficulties to dilate the pupil	increased risk of postoperative posterior capsule opacification
	risk of diabetic retinopathy exacerbation or developing diabetic macular edema

## Supplementary Materials

The following are available online at https://www.mdpi.com/2077-0383/8/5/716/s1, Supplement flie S1: Search strategy.
