# Supplementary material for: Diabetes and Phacoemulsification Cataract Surgery: Difficulties, Risks and Potential Complications
Source: J Clin Med. 2019 May 20;8(5):716. doi: 10.3390/jcm8050716 (PMC6572121; doi:10.3390/jcm8050716)
Supplement: Supplementary file 1 [file jcm-08-00716-s001.pdf]

Supplement 1. Search strategy

Literature searches of the PubMed and Web of Science databases were conducted in October 31, 2018; the search strategies are as follows. Specific limited update searches were conducted after October 31, 2018. Reference lists of the included studies were also considered as a source of publications.

A.1. PubMed Search (Publication Date 1/10/11–10/31/2018)
((“diabetes”[Title]) OR (“diabetic”[Title])) AND ((“cataract surgery”[Title]) OR (“phacoemulsification”[Title])).
147 references.

A.2. Web Of Science Search (Publication Date 1/10/11–10/31/2018)

(TI=(diabetes) OR TI=(diabetic)) AND (TI=(cataract surgery) OR TI=(phacoemulsification))
Indexes=SCI-EXPANDED, SSCI, A&HCI, CPCI-S, CPCI-SSH, BKCI-S, BKCI-SSH, ESCI, CCR-EXPANDED, IC Timespan=All years
333 references.
